# Clinical Utility of D-Dimer for Rule-Out or Rule-In of Venous Thromboembolism in Syncope

**DOI:** 10.1007/s12265-022-10306-0

**Published:** 2022-08-30

**Authors:** Patrick Badertscher, Jeanne du Fay de Lavallaz, Angelika Hammerer-Lercher, Christian Mueller, for the BASEL IX Investigators, Tobias Zimmermann, Pedro Lopez-Ayala, Thomas Nestelberger, Danielle M. Gualandro, Òscar Miró, Dagmar I. Keller, F. Javier Martin-Sanchez, Franz Bürgler, Martin Than, Velina Widmer, Michael Christ, Louise Cullen, Salvatore Di Somma, W. Frank Peacock, Nicolas Geigy, Michael Kühne, Michael Freese, Emilio Salgado, Gemma Martinez-Nadal, Carolina Isabel Fuenzalida Inostroza, Juan Pablo Costabel, José Bustamante Mandrión, Imke Poepping, Katharina Rentsch, Arnold von Eckardstein, Andreas Buser, Jaimi Greenslade, Jens Lohrmann

**Affiliations:** 1 Cardiovascular Research Institute Basel (CRIB) and Department of Cardiology, University Hospital Basel, University of Basel, Petersgraben 4, CH-4031 Basel, Switzerland; 2 GREAT Network, Basel, Switzerland; 3 Department of Laboratory Medicine, Kantonsspital Aarau, Aarau, Switzerland

**Keywords:** Syncope, Pulmonary embolism, Diagnostic testing

## Abstract

Fig. 1 Diagnostic performance of D-dimer using two different assays in patients presenting with syncope. A Left: Receiver-operating characteristic curves quantifying the diagnostic performance of Innovance® D-dimer (blue) and hs-Loci-Innovance® D-dimer (red) for the diagnosis of venous thromboembolism (VTE). Right: Clinical application of D-dimer using the 2-level Wells-score with age-adjusted^1^ or fixed cutoffs versus the YEARS-algorithm with probability-adjusted cut offs^2^. **B** Left: Specificity for different cufoffs of Innovance® D-dimer (blue) and hs-Loci-Innovance® D-dimer (red) for the diagnosis of venous thromboembolism (VTE). Right: Percentage of patients ruled-in and correctly identified VTE patients for different cutoffs of Innovance® D-dimer (blue) and hs-Loci-Innovance® D-dimer (red). ^1^In patients 50 years or younger, D-dimer concentration < 0.5 mg/l was considered negative. For patients older than 50 years, we used the formula: age in years divided by 100. ^2^YEARS-algorithm: assessment of only three items from the Wells-score (clinical signs of deep vein thrombosis, hemoptysis, pulmonary embolism the most likely diagnosis) and using a D-dimer test threshold of 0.5 mg/l in presence, and 1.0 mg/l in absence of one of the YEARS-items 
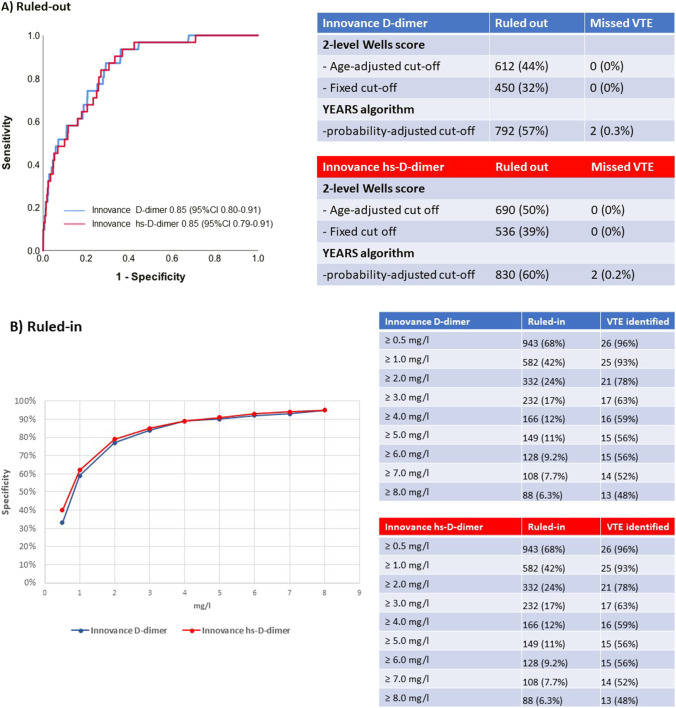

Syncope is a common reason for patient presentation to the emergency department (ED). Its differential diagnosis often is challenging and includes benign vasovagal, but also acute life-threatening cardiovascular causes such as pulmonary embolism (PE) [1]. As syncope is a rather uncommon presentation of PE [1–3], it is largely unknown whether D-dimer concentrations, which are recommended in suspected PE and low-to-intermediate pre-test-probability [4], can also be applied in patients presenting with syncope.

We aimed to address these uncertainties in a large diagnostic multicenter study prospectively enrolling unselected patients presenting with syncope to the ED (NCT01548352) [1]. Patients were enrolled prospectively from May 2010 to February 2017. Patients with ongoing anticoagulation therapy and with missing blood samples were excluded. D-dimer was measured in a blinded fashion using two different assays in a central laboratory: Innovance® D-dimer [5]and Innovance® Loci-high-sensitivity D-dimer (Siemens Healthcare, New York, USA). The presence of venous thromboembolism (VTE = PE or venous thrombosis) at presentation and/or during 720 days follow-up was centrally adjudicated by independent cardiologists according to guidelines as described in detail elsewhere [4]. In brief, patients were determined to not have an index PE if none of three assessments provided evidence of VTE: (A) the initial clinical work-up possibly including computed-tomography pulmonary angiography or ventilation/perfusion-scanning; (B) the initial study-specific work-up including Wells score and D-dimer in all patients; (C) 720-day clinical follow-up. To maximize sensitivity for VTE, all VTE and all cardiovascular deaths without clear alternative causes occurring during 720-day follow-up were considered clinically missed VTE at index-presentation. Patients were excluded if on oral anticoagulation.

Three endpoints were evaluated: First, the diagnostic accuracy of D-dimers for PE if measured in unselected patients presenting with syncope to the ED. Second, the diagnostic performance of applying D-dimer within the three most widely used algorithms for the rule-out of PE: (a) 2-level Wells-score using age-adjusted, (b) fixed cutoffs, and (c) the YEARS-algorithm with only three items of the Wells-score (clinical signs of deep vein thrombosis, hemoptysis, PE the most likely diagnosis) and using a D-dimer threshold of 0.5 mg/L in presence, and 1.0 mg/L in absence of one of the YEARS-items (4). Third, the specificity for different D-dimer cutoffs to rule-in VTE.

Among 1396 eligible patients, mean age was 68 years, 42% were women, median duration of follow-up was 751 days (IQR, 722–873 days), and 19 patients were adjudicated to have VTE at index presentation and 31 patients (2.2% [95%CI 1.5–3.1%]) to have VTE at index or during follow-up. D-dimer concentration was higher in syncope due to VTE versus those from other causes (Innovance® 6.86 mg/l versus 0.78 mg/l, p < 0.001). The diagnostic accuracy of D-dimer for VTE as quantified by the area under the receiver-operator-characteristics curve was high: Innovance® 0.85 (95%CI 0.80–0.91, Fig. 1). In these patients, when using a fixed cutoff of D-dimer concentration < 0.5 mg/L, 32% of patients (n = 450), and when using age-adjusted cutoffs, 44% of patients (n = 612), could safely be ruled out (without missing any patients with VTE at the index presentation or during FU). When using the YEARS algorithm with pre-test-probability adapted cutoffs, 57% of patients (n = 792) could be ruled-out, thereby missing 2 non-fatal PE cases (1 at Index presentation, 1 during FU after 55 days). Internal validation using a second D-dimer-assay (Innovance® Loci-high-sensitivity) confirmed these findings (Fig. 1A). Regarding VTE rule-in, the specificity for the diagnosis of VTE increased with increasing D-dimer concentrations (Fig. 1B). Using a cutoff of 8 mg/l, specificity for VTE was 95% (95%*CI* 93–96%).

Three insights of major clinical relevance evolved from these analyses. First, D-dimer concentrations were significantly higher in patients with syncope due to adjudicated VTE versus those with other causes, providing high diagnostic accuracy for VTE. Second, using D-dimer concentration within one of the three widely used PE rule-out algorithm with their established cutoffs provided high safety. Third, D-dimer concentrations of 8 mg/L or higher have a specificity of 95% and should be considered also as part of rule-in pathways for VTE.

A limitation of this study is that not all patients underwent definitive VTE-imaging during the index evaluation, but all patients underwent clinical follow-up for up to 720 days. We would expect clinically relevant PE accounting for syncope to manifest itself during 720-day FU period; thus, the diagnosis of PE was infrequently missed.

In conclusion, D-dimer provides high accuracy for the detection of VTE in unselected patients presenting with syncope to the ED, thereby providing high clinical utility for rapid rule-out with excellent safety as well as rule-in of VTE.
